# Nitrogen Assimilation in the Highly Salt- and Boron-Tolerant Ecotype *Zea mays* L. Amylacea

**DOI:** 10.3390/plants9030322

**Published:** 2020-03-04

**Authors:** Teresa Fuertes-Mendizábal, Elizabeth Irica Bastías, Carmen González-Murua, Mª Begoña González-Moro

**Affiliations:** 1 Departamento de Biología Vegetal y Ecología, Facultad de Ciencia y Tecnología, Universidad del País Vasco/EHU, Apdo. 644, E-48080 Bilbao, Spain; teresa.fuertes@ehu.eus (T.F.-M.); carmen.gmurua@ehu.eus (C.G.-M.); 2 Departamento de Producción Agrícola, Facultad de Ciencias Agronómicas, Universidad de Tarapacá, Arica 1000000, Chile; ebastias@uta.cl

**Keywords:** amino acids, glutamine synthetase, nitrate reductase, proline

## Abstract

The Lluta Valley in Northern Chile is an important agricultural area affected by both salinity and boron (B) toxicity. *Zea mays* L. amylacea, an ecotype arisen because of the seed selection practiced in this valley, shows a high tolerance to salt and B levels. In the present study the interaction between B and salt was studied after 20 days of treatment at low (100 mM) and high salinity (430 mM NaCl), assessing changes in nitrogen metabolites and in the activity of key nitrogen-assimilating enzymes. Under non-saline conditions, the presence of excessive B favored higher nitrate and ammonium mobilization to leaves, increasing nitrate reductase (NR) activity but not glutamine synthetase (GS). Thus, the increment of nitrogen use efficiency by B application would contribute partially to maintain the biomass production in this ecotype. Positive relationships between NR activity, nitrate, and stomatal conductance were observed in leaves. The increment of major amino acids alanine and serine would indicate a photoprotective role of photorespiration under low-salinity conditions, thus the inhibition of nitrogen assimilation pathway (NR and GS activities) occurred only at high salinity. The role of cytosolic GS regarding the proline accumulation is discussed.

## 1. Introduction

The restriction in plant growth and productivity caused by salinity is especially severe in arid and semi-arid regions. The Lluta Valley in Northern Chile is a region where annual precipitation is lower than 1.1 mm and high levels of boron coming from alluvial deposits are present together with others salts originated from Cretaceous shales. Due to these environmental factors, the agronomic productivity in the region is limited to a bunch of relatively salt tolerant ecotypes. At present, amylacea maize pre-Columbian ecotype represents one of the main crops for this region due to its high productivity [1].

High concentrations of salts may reduce plant growth and crop productivity by water deficit, nutrient ion imbalance, and ion toxicity in the cell, or a combination of any of these adverse factors [2,3,4]. Salinity is also reported to decrease nitrate uptake and to affect nitrogen metabolism; firstly, due to a direct competition between Cl^−^ and NO_3_ for uptake at both paths, the plasmalemma and tonoplast, and also in the processes of transport and translocation of nitrate loaded into the xylem cells [5,6], since nitrate transport can be mediated by chloride channel (CLC) family [7,8,9]. Secondly, salts can affect protein–lipid interactions at membrane level, altering the plasmalemma integrity [10]. The activity of nitrate reductase (NR) in leaves is largely dependent on the nitrate flux from the roots [5,11], which can be affected severely by saline stress [12], since frequently water flux to the aerial parts is reduced due to the stomatal closure. Thus, salinity may have severe consequences for nitrate assimilation in photosynthetic organs.

In higher plants, glutamine synthetase (GS) plays a pivotal role in ammonium assimilation coming from the primary assimilation of nitrate, photorespiration, and catabolic processes into nitrogenous organic compounds, acting together with the glutamate synthase (Fd-GOGAT) in the GS/GOGAT cycle. Some studies have demonstrated that saline stress reduced the GS activity in soybean roots [13], rice [14,15], tomato [6,16], wheat [17], and *Catherantheus roseus* [18]. However, opposite results were also observed in plants of cowpea [19], rice [20], foxtail millet plants [21], or barley roots [22]. In general terms, the activities of the enzymes involved in the ammonium assimilation appear sensitive to salt presence in glycophytes, but the effects of salinity on nitrogen metabolism are highly complex [23]. Probably, the responsiveness to salinity depends on many other factors such as plant species, nitrogen fertilization, stress intensity, plant age, and also plant tissue. Maintaining a good capacity to assimilate nitrogen is a necessity for crop plants growing under saline conditions in order to achieve an optimum productivity. The nitrogen-assimilating activity will determine the capacity of the plant to synthesize glutamate and glutamine as primary amino donors, as well as other nitrogenous compatible solutes, such as proline or glycine-betaine, essential to cope with saline stress. Thus, maintaining the N assimilating capacity results is of special interest for the selection of salt-tolerant crop cultivars.

Boron is an essential micronutrient required for vascular plants, being involved in different processes such as vegetative growth, synthesis and structure of the cell wall, lignification, plasma membrane integrity and function, phenolic metabolism, seed development and sugar transport, among others [24,25,26]. Previous results by our research group showed that the availability of B mitigated in part the negative effects of salinity on amylacea ecotype, allowing the recovery of K^+^ levels (homeostasis), the maintenance of the membrane integrity and the elasticity of the cell wall [1]. To date, although there is no convincing evidence for a direct effect of B on nitrate assimilation, it seems to be involved in one way or another in nitrogen metabolism [24,26,27,28]. The involvement of boron on the expression of genes related to nitrogen metabolism [28,29], oxidative stress, B uptake and cell wall [30] was reported. Besides, its possible role as cellular signal through the interaction with transcription factors [30] or even with calcium-mediated pathways [25] was considered. To the best of our knowledge, little is known about the effect of B on the activities of nitrogen-assimilating enzymes in important food crops, such as maize [31]. For this reason, the study of nitrogen metabolism in a highly boron-tolerant cultivar, such as amylacea, may be relevant, especially under saline conditions.

The present study was carried out to establish the behavior of nitrogen metabolism in a highly boron-and salt-tolerant cultivar, as *Zea mays* L. amylacea “Lluta”. The interaction between B and salt was studied by assessing changes in nitrogen metabolites and in activities of key nitrogen-assimilating enzymes. Knowing the effect of salinity and boron on nitrogen metabolism in this cultivar will shed new understanding about how biochemically and physiologically this ecotype succeed under extreme saline conditions, maintaining an optimum productivity in the Lluta desert region. The study was carried out on amylacea ecotype after 20 days of treatment at low (100 mM NaCl) and high (430 mM NaCl) salinity and at two B levels (20 and 40 mg Kg^−1^).

## 2. Results

### 2.1. Plant Biomass Production

**Figure 1:**
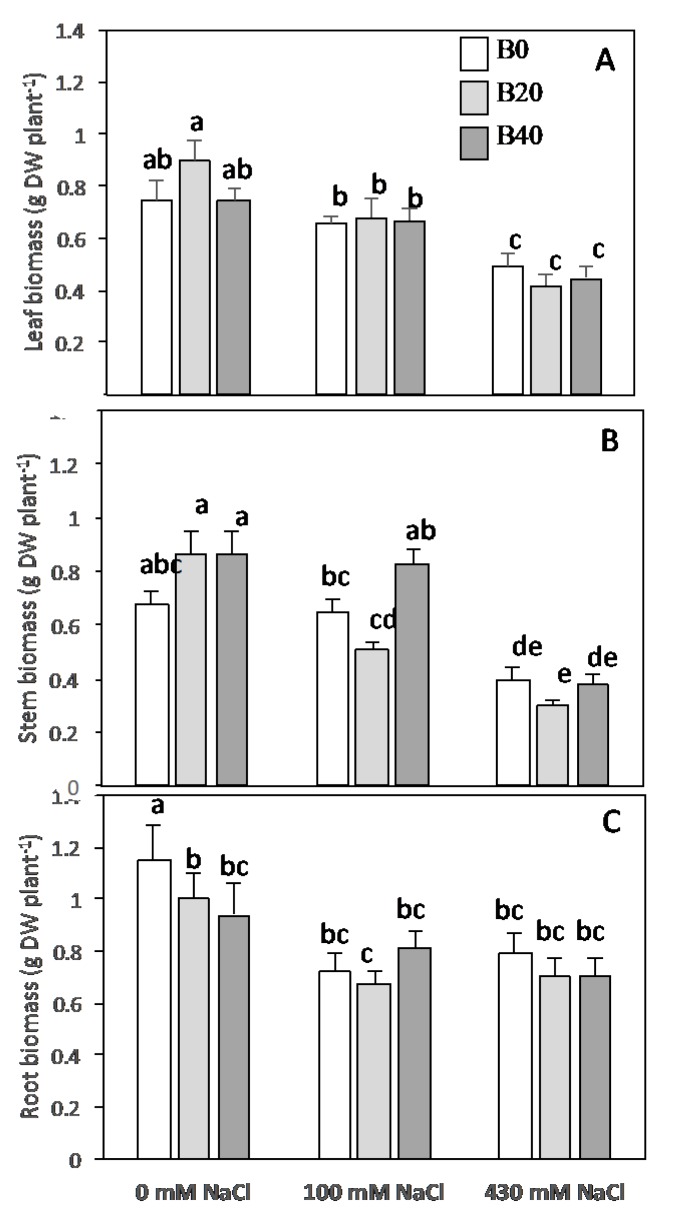
Leaf (**A**), stem (**B)**, and root (**C**) dry weight of *Zea mays* L. amylacea after 20 days of treatment with NaCl (0 mM; 100 mM; 430 mM) and H_3_BO_3_ (B0, control; B20, 20 mg kg^−1^; B40, 40 mg kg^−1^). Values represent the mean ± SE of three independent experiments. Bars with the same letter are not significantly different according to LSD at level of *p* < 0.05.

Partial variance (η^2^) is used to describe the size effect of the relationship between two variables of study [32]. As shown in Table 1, salinity significantly affected plant biomass accumulation to a higher extent than boron, as partial η^2^ values indicated that most of the variability of plant growth was due to salinity. Thus, biomass, expressed in terms of leaf, stem, and root dry matter accumulation, decreased in presence of NaCl (Figure 1). Reductions of leaf biomass in plants grown at 100 and 430 mM NaCl were statistically significant with decreases of 15% and 43%, respectively, and so for stem, which biomass reductions respect to control were even higher, 18% and 55% for low and high salinity treatments, respectively. In contrast, application of B levels considered toxic for the most crops (20 and 40 mg kg^−1^) did not significantly alter the growth of the maize amylacea cultivar. Under non-saline conditions, only a slight decrease was observed in root dry biomass by the addition of extra boron, whereas under salt conditions the different plant organs achieved the same yield biomass, regardless of the boron application.

### 2.2. Nitrate, Ammonium, Amino Acids, and Soluble Protein

**Figure 2:**
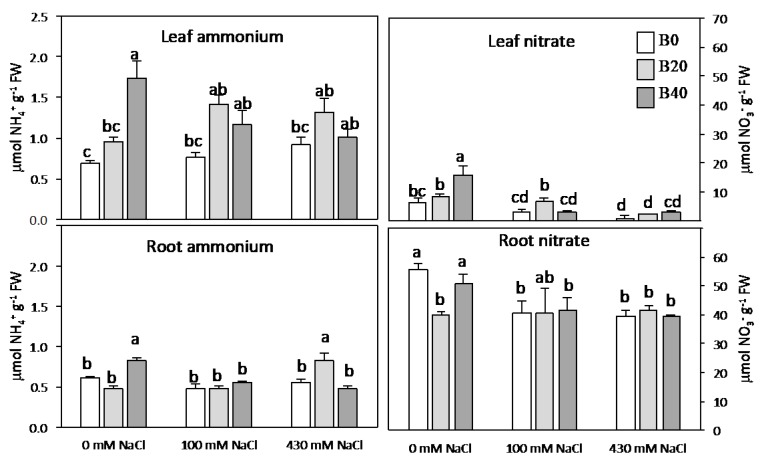
Content of nitrate and ammonium in leaf (top) and root (bottom) tissues of *Zea mays* L. amylacea, after 20 days of treatment with NaCl (0 mM; 100 mM; 430 mM) and H_3_BO_3_ (B0, control; B20, 20 mg kg^−1^; B40, 40 mg kg^−1^). Values represent the mean ± SE of three independent experiments. Bars with the same letter are not significantly different according to LSD at level of *p* < 0.05.

**Figure 3:**
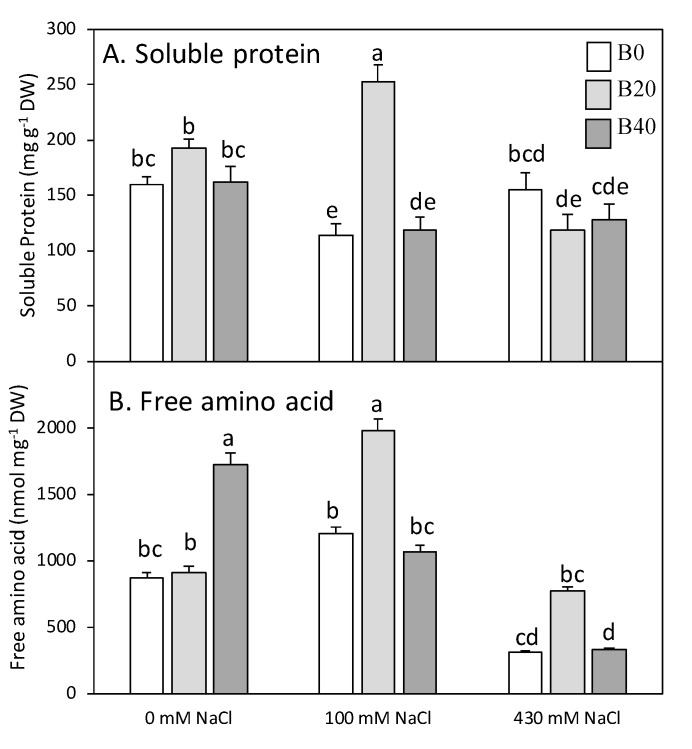
Leaf soluble protein (**A**) and free amino acid (**B**) contents of *Zea mays* L. amylacea after 20 days of treatment with NaCl (0 mM; 100 mM; 430 mM) and H_3_BO_3_ (B0, control; B20, 20 mg kg^−1^; B40, 40 mg kg^−1^). Values represent the mean ± SE of three independent experiments. Bars with the same letter are not significantly different according to LSD at a level of *p* < 0.05.

**Figure 4:**
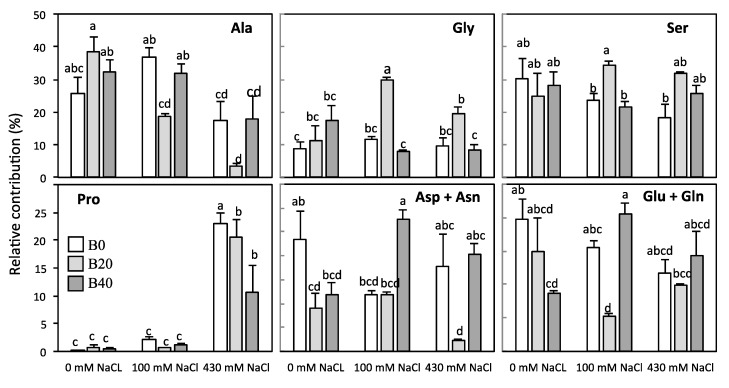
Relative contribution of alanine (Ala), glycine (Gly), serine (Ser), proline (Pro), aspartate + asparagine (Asp + Asn), and glutamate + glutamine (Glu + Gln) in leaves of *Zea mays* L. amylacea after 20 days of treatment with NaCl (0 mM; 100 mM; 430 mM) and H_3_BO_3_ (B0, control; B20, 20 mg kg^−1^; B40, 40 mg kg^−1^). Values represent the mean ± SE of three independent experiments. Bars with the same letter are not significantly different according to LSD at level of *p* < 0.05.

Nitrate content was considerably higher in root than in leaf tissue (Figure 2). On the contrary, leaf and root ammonium contents were similar when no extra B was applied, whatever the NaCl treatment was (Figure 2). Attending to the effect size of each factor (Table 1), boron affected only ammonium content in leaf, whereas both boron and salinity, and their interaction, changed leaf nitrate content (Figure 2). Under non-saline conditions, excess B doubled the content of nitrate and ammonium in leaves. On the contrary, under saline conditions the nitrate content drastically decreased in leaves, whereas ammonium content was maintained showing an increment with the application of extra B. Both factors, B and salinity, as well as their interaction, affected significantly the content of total free amino acids and soluble protein, as shown by partial η^2^ values, although to a greater extent in the case of total free amino acids (Table 1). Thus, strong increases (>80%) were observed in free amino acid content for B40 applied under non-saline conditions, and for B20 under both salt levels (100 and 430 mM) (Figure 3B). The application of B20 under 100 mM NaCl was the treatment that accumulated the highest amino acid and soluble protein contents (Figure 3). At high salinity, only the pool of free amino acid drastically decreased. The most-abundant free amino acids in leaf under non-saline conditions were alanine (25–40%), serine (23–30%), glycine (10–18%), glutamate + glutamine (10–24%), and aspartate + asparagine (7–20%), whose levels accounted for, at least, 84% of the total free amino acid pool (Figure 4). Other amino acids, such as valine, tyrosine, and phenylalanine together represented approximately 6% of the overall amount (data not shown), while proline showed minimal values in leaf tissue of non-salinized plants (Figure 4). Relative proportions of major amino acids and proline pattern changed significantly in leaves of B- or salt-treated plants. However, the size of the effect according to partial η^2^ values was higher for salinity factors in all amino acids (Table 1). Under non-saline conditions, the presence of B tended to rise the relative contributions of alanine and glycine, meanwhile those of glutamate + glutamine and aspartate + asparagine significantly decreased (Figure 4). Under salinity glycine and serine increased if moderate B levels (B20) were applied, while alanine decreased. Glutamate + glutamine or aspartate + asparagine pools also decreased with B20, the former under low salinity and the latter under high salinity. Nonetheless, at high saline conditions, the most noticeable changes were the high occurrence of proline and the depletion of alanine contribution. The former increased strongly, accounting a maximum of 23% of the total leaf amino acid pool in the absence of B at high salinity, whereas the presence of B partially counteracted the accumulation of Pro.

Soluble protein content tended overall to decrease with salt stress, with an average of 28% at low salinity, except when B20 was present, and between 22–39% at high salinity level when B was applied (Figure 3A).

### 2.3. Activity and Expression of Marker Enzymes for N Assimilation

**Figure 5:**
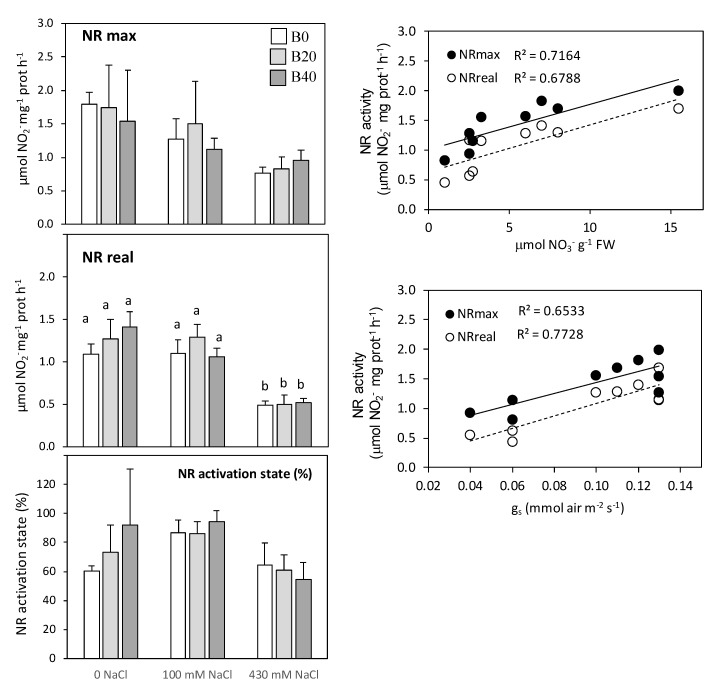
Maximum nitrate reductase (NRmax) and real nitrate reductase (NRreal) and nitrate reductase (NR) activation state in leaves of *Zea mays* L. amylacea after 20 days of treatment with NaCl (0 mM; 100 mM; 430 mM) and H_3_BO_3_ (B0, control; B20, 20 mg kg^−1^; B40, 40 mg kg^−1^). Values represent the mean ± SE of three independent experiments. Bars with the same letter are not significantly different according to LSD at a level of *p* < 0.05. On the right side: relationships between NR and stomatal conductance (g_s_) (right top) and NR and nitrate content in leaves (right bottom) of the amylacea ecotype of *Zea mays* L. amylacea.

Nitrate assimilation depends on nitrate reductase activity and glutamine synthetase in leaves. The addition of B40 (40 mg kg^−1^) under non-saline conditions tended to increase approximately 30% of leaf NR activity (Figure 5, left). The maximum NR activity assayed in the presence of EDTA (NR_max_) should reflect the maximum amount of functional protein, whereas the activity assayed in presence of Mg^2+^ (NR_real_) represents the activity of the dephosphorylated protein. At low salinity, the inhibition of NR activity was minimal whereas at high salinity the NR_max_ activity decreased by about 30%, and even a greater decrease (60%) was detected in the NR_real_ under these conditions. Thus, leaf NR enzyme presented high activation states for control non-saline and low salinity conditions, with values ranging from 60 to 90%, whereas at high salinity, the activation state of the enzyme was reduced to 55–60%. Furthermore, a highly linear correlation between NR activity, either NR_real_ or NR_max_, and nitrate content in leaves was observed (R^2^ > 0.678), showing that increasing contents of nitrate in leaves were accompanied by increases in NR_max_ and NR_real_ activities (Figure 5, right). Similarly, a positive linear correlation was observed between NR activity and stomatal conductance (*g*_s_) (Figure 5, right; R^2^ ranging between 0.653 and 0.772).

**Figure 6:**
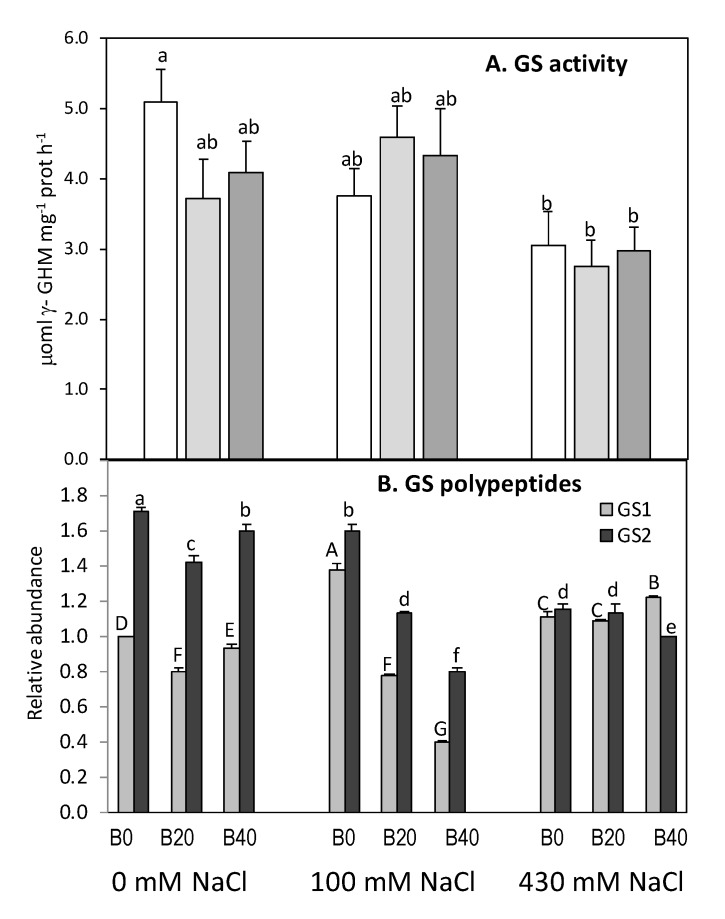
Glutamine synthetase activity (**A**) and relative abundance of glutamine synthetase (GS) polypeptides (**B**) in leaves of *Zea mays* L. amylacea after 20 days of treatment with NaCl (0 mM; 100 mM; 430 mM) and H_3_BO_3_ (B0, control; B20, 20 mg kg^−1^; B40, 40 mg kg^−1^). The relative amounts of GS polypeptides were calculated following densitometric scanning of the Western blot and expressed as fold change in relation to the amount of GS1 (cytosolic GS polypeptide) in control plants (0 mM NaCl and B0). Similar protein content (20 mg) was loaded in each lane. Values represent the mean ± SE of three independent experiments. Bars with the same letter are not significantly different according to LSD at level of *p* < 0.05.

The addition of extra B under non-saline condition tended to deplete GS activity in leaves, between 20–27% (Figure 6), although non-significantly. Under low salinity, no significant changes were observed in GS activity, and only under high saline conditions GS activity was reduced by 31%. Therefore, as partial η^2^ values showed (Table 1), only salinity exerted a significant effect on GS activity. However, both salinity and B significantly affected GS polypeptides, cytosolic (GS1) and chloroplastic (GS2) isoenzymes, monitored by immunoblotting. Under both non-saline and low salinity conditions, GS2 was the predominant isoform in the leaves of this ecotype. The application of B levels considered toxic for most crops slightly depleted both GS polypeptides under non-saline conditions. However, at low salinity conditions, the presence of GS1 increased significantly when no extra B was supplied and decreased with B40, while GS2 decreased with B addition. At high salinity conditions, the abundance of GS1 increased, with a concomitant decrease of the presence of GS2 polypeptide, so that both polypeptides were equally abundant, regardless the presence of B.

## 3. Discussion

### 3.1. Boron Improves N Assimilation

Boron-tolerant cultivars of different crops have been identified, this tolerance being associated with a reduced accumulation of boron in tissues and related to an active efflux of boron out root cells [33,34]. Previous studies revealed that amylacea ecotype shows a strategy related to translocation of boron to aerial parts, meanwhile B improved photosynthetic activity and water relations [1]. Accordingly to present results, B also stimulates N metabolism in *Zea mays* amylacea. The additional supply of B favored a higher nitrate mobilisation from roots to leaves, as can be deduced from the lower o similar contents of nitrate in roots parallel to the increment of this element in leaves (Figure 2). This effect could be mediated by the requirement of B at a transcriptional level, since it has been reported for tobacco that B deprivation limited the transcription of the NRT2 gene, involved in the transport of nitrate, or at the level of root–plasma membrane H-ATPase transcript [26,28,31]. In amylacea ecotype, the activating effect of B on nitrogen assimilation may be the result of different factors, such as a facilitation of nitrate transport to the leaf (Figure 2) [35], a direct stimulation of the synthesis of enzymatic proteins or a direct influence of B on enzymatic activities [36,37]. Thus, a B-mediated increasing de novo synthesis of NR protein has been described in oilseed rape [38] and tobacco [27]. Under non-saline conditions, the slight decline of GS activity (Figure 6) observed with the application of boron was not limiting for the nitrogen-assimilation process, as soluble protein and biomass production were maintained or even increased. These results relatively agree with those observed in tobacco leaves [27,35], and tomato leaves [37], since a stimulation of NR, GS, and other nitrate-assimilation enzymes, such as nitrite reductase and GOGAT, also took place in presence of B. In the same line of evidence, the involvement of B on nitrogen assimilation was also reported in boron deficient plants, in which the inverse occurred [36,39]. Thus, the adaptation of amylacea ecotype to high boron levels in Lluta Valley regarding the N metabolism could be considered as a general response, similar to that observed in other non-adapted species [27,35,37].

The accumulation of ammonium and amino acids in presence of B in amylacea ecotype would indicate that eventually boron per se improves the nitrate assimilation and favors the N metabolism. The increase in the amount of soluble protein (Figure 3) with low B level would favor the positive effect of boron on the CO_2_ photosynthetic assimilation, a fact previously reported for this ecotype [1]. Based on growth data and changes in N metabolism presented in this study, this ecotype shows a high adaptation to B, since, as mentioned above, this ecotype not only survives to B concentrations considered toxic for most cultivars, but also maintains biomass production and protein contents in leaves. Therefore, the better efficiency of N use under the presence of B would be one aspect that contributes also to the better performance of this ecotype, despite the boron levels applied being commonly toxic for most crops.

### 3.2. Under Low Salinity N Assimilation Exceeds Plant Demand

Saline stress affects the activity of enzymes involved in ammonium assimilation in plants, although the effect differs depending on the plant species and tissue [6,40], as well as on variations of nutritional factors [22]. The concentration of nitrate in tissues is the balance between the rate of nitrate uptake and its reduction. In this sense, nitrate transport can be mediated by chloride channel (CLC) family [7,8], acting chloride as nitrate antagonist and decreasing its loading into the xylem due to competence. This fits with the idea that the sensitivity of different species to salt is related to the sensitivity of their nitrate uptake systems to chloride [41]. Thus, indirectly chloride can inhibit NR activity o may potentially have a direct effect on the synthesis, degradation, or denaturation of NR protein, therefore modifying the amount of NR protein, as it was observed in leaves of maize and wheat [12,42]. NR activity measured in presence of EDTA (NR_max_) is considered a reflection of the total amount of NR protein, whereas NR_real_ reflects the activity of the dephosphorylated activated protein [11]. The ratio NR_real_/NR_max_, expressed as percentage, is considered its activation state. Amylacea ecotype keeps a high activation state of NR (above 75%) in comparison with other maize cultivars [12,43], even under low salinity conditions (Figure 5); only at high salinity the NR_real_ depleted, but still maintaining a considerable NR activation (55%), which would point out a lower availability of nitrate to the enzyme (Figure 2) [6]. Alternatively, the presence of a less active form would indicate a higher phosphorylation state (Figure 5), which would be provoked by a decrease in NR protein content [11]. In this sense, our results also suggest that NR protein is regulated by the availability of NO_3_^−^ content in leaves under salinity (Figure 5) as previously reported [11,13], probably due to a low NO_3_^−^ loading into the root xylem and/or to a reduced nitrate translocation to shoots [5,12]. Excess Na^+^ and Cl^−^ accumulated in tissues could have a direct influence on the nitrate transport through cell plasmalemma or from the vacuole to the cytoplasm [4,6,12], contributing to lower availability of nitrate in leaf cells. This would occur especially at conditions of low salinity, where no stomatal closure was still observed, and therefore high salt levels were accumulated [1]. However, at high salinity levels the stomatal closure would limit the nitrate flux to aerial organs as deduced by decreased nitrate contents (Figure 2 and Figure 5). The positive correlation between NR activity and nitrate content in leaves of amylacea (Figure 5) reflects the idea that nitrate availability would be the most-decisive factor that regulates NR expression, translation, protein content, and its activation in higher plants [5,11,12,42]. Nevertheless, despite the considerable diminution of nitrate content in amylacea leaf with saline conditions, its level seemed sufficient to maintain the NR activity in order to guarantee nitrogen assimilation; therefore, nitrate reduction process would reveal a good adaptation to salinity conditions in this ecotype, in contrast to other maize cultivars or varieties in which NR activity and growth are considerably affected at salt levels of 200 mM [12,44]. Therefore, we can conclude that nitrate reduction would not be limiting for the growth of amylacea at low salinity, since at the twentieth day of exposure to salinity no effect on NR was observed, whereas an inhibition of 40% was already evident in biomass production (Figure 1). These results are in accordance with the fact that nitrate reduction exceeds the plant nitrogen demand [12,42].

Cytosolic GS plays a role in primary nitrogen assimilation in root, while in leaves, different physiological roles have been proposed for GS1 and GS2 in ammonium assimilation. GS1 participates in glutamine biosynthesis along the entire plant development cycle and reassimilates the ammonia released from remobilization of leaf proteins during aging and natural senescence [45,46,47,48], or even in response to salt stress [49]. Plastidic GS2 seems to play a dual a role; in early stages of plant development GS2 reassimilates photorespiratory ammonium, released in a much lower quantity in C4 plants as maize compared with C3 plants; it also assimilates the ammonia resulting from nitrate reduction, a process that occurs specifically in mesophyll cells in C4 plants [47]. The results of amylacea ecotype showed a high tolerance of its GS activity to saline conditions, corroborated by the fact that still 59% GS activity remained in response to high salt levels and boron absence (Figure 6). The decrease of total leaf GS activity (28–41%) in amylacea under saline conditions was primarily due to the down-regulation of the chloroplastic polypeptide (GS2), while the relative accumulation of GS1 increased slightly (Figure 6). These results agree with those reported for plants of potato [40] and tomato [6]. The increased relative expression of GS1 may reflect an attempt by the shoots to overcome the salt toxicity imposed, thus contributing to maintain the GS activity and ammonium assimilation, and probably plays a role in regulating proline production, mainly at high salinity [21,49]. In fact, increased GS activity has been suggested as a biochemical adaptation for salt-tolerant species [50,51] and even within the same species [14].

Changes in amino acid content at low salinity have been described as an adaptive response in order to maintain the intracellular osmotic pressure during the saline stress in maize [12], wheat [42] and spinach [52]. Amino acids can contribute to osmoprotective processes, serve as N source, mitigate oxidative stress by scavenging radicals [53], or play a role in photorespiratory pathways [54,55]. The relative decrease detected in Asp + Asn and Glu + Gln contents at salinity would suggest that amino groups are derived to the synthesis of other amino acids, like Ser, Ala, Gly, but particularly to Pro [54,56]. Ala and Gly could be supporting the photorespiration pathway, as a protective physiological process under salinity. The predominance of Ala amongst the major amino acids in the amylacea ecotype agrees with data available for other maize hybrids [55,56]; the high occurrence of Ala would indicate its role in maize metabolism, perhaps maintaining the transamination reaction to Ser and Gly, as could be deduced from the mirrored changes of the first versus Ser and Gly (Figure 4).

Under high-dosage of NaCl stress (430 mM NaCl), the physiological state of the amylacea ecotype was quite different. The strong diminution of total free amino acids was, in part, expected, due to the depletion of nitrogen-assimilating activities (NR and GS) observed when a high salt concentration was present in the growth medium. We can assume, based on its highly relative presence, that Pro could play a key role in osmoprotection at high salinity. Pro presence is known to rise in several plant tissues in response to a wide variety of abiotic stresses, mainly water and saline stress [23,57]. Moderate accumulation of Pro is a characteristic metabolic response to osmotic stress in glycophytes and it has been previously reported to accumulate in maize under saline stress [12,44]. In amylacea ecotype, the drastic increment of Pro (representing up to 24% of the total soluble amino acids) observed only at high salinity was produced at the expense of Ala pool. Since Pro is an amino acid belonging to the glutamate family, the accumulation of high levels of Pro will be only possible if a sufficient amount of its precursor Glu is provided. In this sense, depletion of Glu levels (Figure 4) indicated that it probably was being channeled to the synthesis of Pro under saline condition [52,58]. Ala can take part in transamination reactions, and its decrease would indicate an essential role in the synthesis of Pro at high salinity in amylacea ecotype. Although the role of Pro under salt stress is still, nowadays, a matter of discussion, the accumulation of Pro suggests a protective role under ionic stress, acting either as osmoregulator or osmoprotectant [23]. The relatively high content of soluble protein at high salinity indicates that proteolysis process does not occur in a great extent, thus the accumulation of Pro would take place through de novo synthesis as described in some plants subjected to salt stress [59], mainly if glutamate is still provided for its synthesis. In this sense, the remaining GS activity and the role before discussed for GS1 polypeptide would probably contribute to Pro synthesis.

### 3.3. Moderate B Levels Favour Amino Acid Synthesis and Soluble Protein Content Under Saline Conditions

Studies addressing how salt and boron stresses simultaneously affect crops are limited, and their conclusions are contradictory. While some works indicate an increased tolerance to boron under salinity in plants [60,61,62], the contrary has also been documented [63,64,65]. In amylacea ecotype after 20 days of treatment application, overall attending to the changes observed in different biochemical parameters and the partial η^2^ values, we can conclude that the effects of combined salinity and B stress on plant growth attributes are not different to those measured under saline conditions alone. The amylacea variety has been comparatively tested with commercial cultivars Prays-214 and GH-2041, which exhibited severe necrotic injuries after 10 days of exposition to 150 mM NaCl and 20 ppm B [66]. In our study, only a slight interaction between salt and boron was observed in the stem dry matter accumulation at high boron levels; however, there is a positive interaction at the level of nitrogen metabolism between salinity levels and moderate B content (B20), since total amino acids or protein contents are enhanced. The maintenance of pathways for nitrate reduction and ammonium assimilation under low saline levels further would support an increase of photorespiration. Thus, this activation of N metabolism would be fostered by the higher ammonium contents in leaf when B is present, and would favor the synthesis of Gly and Ser, at the expense of Ala. The rise in these photorespiratory amino acids would suggest a photoprotective role of the photorespiration process, as proposed by several authors [52,54]. Alternatively, the stimulation of the photorespiratory pathway could be involved in the production of compatible solutes in plants under saline stress [52]. Interestingly, when B was applied under low salinity, the GS activity was maintained despite the presence of both GS polypeptides depleted. This lack of correlation of GS activity with the expression of its polypeptides at low salinity indicated that B could be mediating post-translational regulatory mechanisms [40] to maintain GS activity. The relative higher presence of GS2 at low salinity would be also concordant with an increased flux of Ser and Gly (Figure 4) through the photorespiratory pathway, in order to reassimilate the photorespiratory ammonium. Amino acid accumulation could indicate potentially, at first sight, proteolysis or biosynthesis inhibition of constitutive proteins under saline stress. However, the relatively high activities of nitrogen reduction and assimilation enzymes (NR, GS) at low salinity coincident, with an increment in soluble protein and free amino acids at 20 mg B Kg^−1^, made us rule out a proteolysis process at low salinity. Under high saline conditions, however, the effect of moderate levels of B is still appreciated, similarly as it occurred at low salinity, on total free amino acids content; and probably related to the higher ammonium levels, and Ser and Gly contents, to maintain the photorespiratory pathway. However, high B (B40) supply made less Pro were necessary, compared to plants treated without extra B or with moderate levels. The fact that B partially reversed leaf Pro contents could be attributed to a lower Na^+^ accumulation in amylacea plants when grew at high salinity [1]. Thus, in the presence of high boron levels, a lower osmoregulatory capacity would be required, and consequently Pro accumulation occurred at a minor extent. Therefore, the high tolerance to hyper-arid conditions of amylacea ecotype, previously confirmed [66], could be due to the adaptation of nitrogen assimilation machinery in this cultivar. Nevertheless, more studies are needed to know the stability and functionality of nitrogen enzymes under salt accumulation and B in tissues of this cultivar. We can conclude that moderate B levels (B20) have in fact a positive effect also on N metabolism in amylacea maize under saline conditions, especially under conditions that allows the nitrate flux to the leaf and therefore nitrate reduction is not limited.

## 4. Materials and Methods

### 4.1. Growth Conditions and Experimental Design

*Zea mays* L. amylacea “Lluta” was germinated in a 1:1 (*v/v*) mixture of perlite and vermiculite and grown in a greenhouse with average day and night temperatures of 25 °C and 18 °C, respectively, at 60–70% relative humidity. Light intensity was set at 350 μmol m^−2^ s^−1^ to provide a 14-h photoperiod. During the first ten days after germination, plants were watered with Hoagland solution containing 20 mM NO_3_-N [67], adjusted to pH 5.5. When maize plants showed the fully expanded second leaf, they were exposed to excess of boron and salt for 20 days. The control treatment consisted of the nutrient solution without salt (0 NaCl) or excess B (B0). The nutrient solution was supplemented with 100 mM NaCl (Low salinity) or 430 mM NaCl (High salinity). The interaction between B and salt was studied also by applying excess B in the form of boric acid (H_3_BO_3_) to the control and low- and high-salinity solutions, in order to obtain 20 (334 µM, B20) and 40 (668 µM, B40) mg B kg^−1^ in the nutrient solution. Nine treatments obtained from the two factors (salt and boron) with six replications were maintained for 20 days.

### 4.2. Stomatal Conductance, Growth Parameters

Measurements of stomatal conductance were carried out on the third fully expanded leaf at the end of the experimental period, as described by our previous work [1]. Afterwards, three replications were harvested, separated into leaves, stem, and roots, and dried to obtain biomass values. Dry matter was determined after oven-drying at 80 °C for 48 h. From the three resting replications, the roots and the third fully expanded leaf were harvested in the first two hours of the light period, frozen instantaneously in liquid nitrogen, and kept at −80 °C until their use for biochemical and metabolic analyses.

### 4.3. Determination of Metabolites: Nitrate, Ammonium, and Amino Acids

For nitrate and free amino acids determinations, fresh and lyophilized root and leaf tissues were respectively extracted successively with a hydroalcoholic series (80% ethanol, 60% ethanol, and Milli-Q water). Nitrate content was measured by the salicylic acid method [68]. For nitrate and ammonium determinations, frozen tissue powder was extracted with 2% sulphosalicylic acid, and determined by the phenol-hypochlorite assay (Berthelot reaction). For amino acids quantification, norleucine was added to each extraction as internal standard, and afterwards amino acids were derivatized with ninhydrin reagent. After ethanol evaporation and resuspension in 2% sulfosalycilic acid, individual free amino acids were analyzed by ion exchange chromatography using an autoanalyzer (Pharmacia Biotech LKB Mod. Biochrom 20, Cambridge, UK).

### 4.4. Enzymatic Assays

Enzymatic activities and protein content were determined for frozen leaf material stored at −80 °C. The NR activity was assayed as the maximal activity in the presence of 5 mM EDTA (NR_max_), and as the real extractable activity in presence of 10 mM MgCl_2_ (NR_real_). NR activation state was estimated as NR_real_/NR_max_, and expressed as a percentage, according to [11]. The extraction for determination of GS enzymatic activity was done as previously described by González-Moro et al. [67], and it was assayed at 30 °C by the method of O’Neal and Joy [69]. Soluble protein content was determined using the method of Bradford [70], with bovine serum albumin as standard protein.

### 4.5. Gel Electrophoresis, the Gel-Staining Procedure, and Protein Blot Analysis

Proteins were extracted from frozen leaf powder in cold extraction buffer, as previously described [38], and separated by 12% SDS-PAGE in a Mini Protean II (Bio-Rad) according to [71]. Equal amounts of soluble protein were loaded per lane (20 μg).

### 4.6. Statistical Analysis

The experimental data were analyzed by ANOVA and differences were compared by the Duncan test with a significance level of *p* < 0.05, using SPSS software, version 11/PC (SPSS 24.0, 2001). The effect size of each factor and their interactions was determined with the use of partial eta-squared, which describes a proportion of variability in a sample associated with an independent variable: η^2^p = SS_effect_/(SS_effect_ + SS_error_), where SS_effect_ is the sum of squares for the effect of interest and SS_error_ is the error term associated with this effect [32]. All experiments described were repeated independently three times with six replications each time. Simple correlation analyses were performed when appropriate.

## 5. Conclusions

Under non-saline conditions, nitrate transport from roots to leaves is stimulated by excess B, accompanied by a slight increase of NR activity, thus confirming the positive effect of this nutrient on the metabolism of N in amylacea maize. The maintenance of nitrate levels in the root indicates that low salinity level (150 mM) does not affect nitrate uptake, but it does affect its transport from roots to the aerial parts. The restricted flux of nitrate to photosynthetic organs due to the stomatal closure observed only at high salinity conditions (430 mM NaCl) affects leaf NR activity and would promote the GS inhibition. Our study confirms previous findings in the sense that GS2 is the isoenzyme primarily down-regulated by salinity. Meanwhile, GS1 accumulation in maize is stimulated in response to salinity, taking over a pivotal role in salt-stressed plants. Thus, these results are in line with a proposed role of GS1 culminating in the production of Gln + Glu as precursors of other amino acids for transamination reactions, mainly for the synthesis of Pro. Under low salinity level, B has also a positive effect on amylacea nitrogen metabolism, stimulating amino acid and protein synthesis; however B is not maintained under high saline conditions, probably because structural damages and a low flux of nitrate to the aerial parts were occurring at high salinity levels.

## Figures and Tables

**Table 1:** Significance and size effect of boron and salinity and their interaction on the different physiological and biochemical variables in leaves of *Zea mays* L. amylacea after 20 days of treatment with NaCl (0 mM; 100 mM; 430 mM) and H_3_BO_3_ (B0, control; B20, 20 mg kg^−1^; B40, 40 mg kg^−1^). * *p* < 0.05, ** *p* < 0.001, *** *p* < 0.001.

	**Leaves DW**		**Stem DW**		**Root DW**		**Ammonium**		**Nitrate**		**Protein**		**Total AA**		**Asp+Asn**		**Ser**	
	**sig**	**partial η^2^**	**sig**	**partial η^2^**	**sig**	**partial η^2^**	**sig**	**partial η^2^**	**sig**	**partial η^2^**	**sig**	**partial η^2^**	**sig**	**partial η^2^**	**sig**	**partial η^2^**	**sig**	**partial η^2^**
**Boron**	ns	0.084	*	0.38	ns	0.13	*	0.237	**	0.504	***	0.419	***	0.521	***	0.374	***	0.563
**Salinity**	***	0.801	***	0.858	***	0.644	ns	0.018	***	0.835	***	0.258	***	0.79	**	0.733	***	0.752
Boron * Salinity	ns	0.258	*	0.495	ns	0.191	ns	0.227	***	0.658	***	0.56	***	0.629	***	0.393	***	0.737
	**Gly**		**Ala**		**Pro**		**Glu+Gln**		**NR real**		**NR max**		**GS activity**		**GS2**		**GS1**	
	**sig**	**partial η^2^**	**sig**	**partial η^2^**	**sig**	**partial η^2^**	**sig**	**partial η^2^**	**sig**	**partial η^2^**	**sig**	**partial η^2^**	**sig**	**partial η^2^**	**sig**	**partial η^2^**	**sig**	**partial η^2^**
**Boron**	***	0.651	**	0.34	***	0.535	ns	0.084	ns	0.032	ns	0.017	ns	0.032	***	0.977	***	0.99
**Salinity**	***	0.664	***	0.892	***	0.772	***	0.679	***	0.485	*	0.173	**	0.288	***	0.988	***	0.988
Boron * Salinity	***	0.803	***	0.665	***	0.637	ns	0.315	ns	0.099	ns	0.044	ns	0.166	***	0.974	***	0.995

## References

[1] Bastías E., González-Moro M.B., González-Murua C. (2004). *Zea mays* L. amylacea from the Lluta Valley (Arica-Chile) tolerates salinity stress when high levels of boron are available. Plant Soil.

[2] Carvajal M., Martínez V., Alcaraz C.F. (1999). Physiological function of water channels as affected by salinity in roots of paprika pepper. Physiol. Plant..

[3] Martinez-Ballesta M.C., Bastías E., Zhu C., Schäffner A.R., González-Moro M.B., González-Murua C., Carvajal M. (2008). Boric acid and salinity effects on maize roots. Response of aquaporins ZmPIP1 and ZmPIP2, and plasma membrane H^+^-ATPase, in relation to water and nutrient uptake. Physiol. Plant..

[4] Parihar P., Singh S., Singh R., Singh V.P., Prasad S.M. (2015). Effect of salinity stress and its tolerance strategies: A review. Environ. Sci. Pollut. Res..

[5] Gouia H., Ghorbal M.H., Touraine B. (1994). Effects of NaCl on flows of N and mineral ions and on NO_3_^−^ reduction rate within whole plants of salt-sensitive bean and salt-tolerant cotton. Plant Physiol..

[6] Debouba M., Gouia H., Suzuki A., Ghorbel M.H. (2006). NaCl stress effects on enzyme involved in nitrogen assimilation pathway in tomato “*Lycopersicon esculentum*” seedlings. J. Plant Physiol..

[7] Wege S., Jossier M., Filleur S., Thomine S., Barbier-Brygoo H., Gambale F., De Angeli A. (2010). The proline 160 in the selectivity filter of the Arabidopsis NO_3_^−^/H^+^ exchanger AtCLCa is essential for nitrate accumulation *in planta*. Plant J..

[8] Zifarelli G., Pusch M. (2010). CLC transport proteins in plants. FEBS Lett..

[9] Zhang H., Zhao F.G., Tang R.J., Yu Y., Song J., Wang Y., Li L., Luan S. (2017). Two tonoplast MATE proteins function as turgor-regulating chloride channels in Arabidopsis. Proc. Natl. Acad. Sci. USA.

[10] Mansour M.M.F., Salama K.H.A. (2004). Cellular basis of salinity tolerance in plants. Environ. Exp. Bot..

[11] Botrel A., Kaiser W.M. (1997). Nitrate reductase activation state in barley roots in relation to the energy and carbohydrate status. Planta.

[12] Abd-El Baki G.K., Siefritz F., Man H.M., Weiner H., Kaldenhoff R., Kaiser W.M. (2000). Nitrate reductase in *Zea mays* L. under salinity. Plant Cell Environ..

[13] Bourgeais-Chaillou P., Pérez-Alfocea F., Guerrier G. (1992). Comparative effects of N-sources on growth and physiological responses of soyabean exposed to NaCl-stress. J. Exp. Bot..

[14] Sahu A.C., Sahoo S.K., Sahoo N. (2001). NaCl-stress induced alteration in glutamine synthetase activity in excised senescing leaves of a salt-sensitive and a salt-tolerant rice cultivar in light and darkness. Plant Growth Regul..

[15] Lin C.C., Hsu Y.T., Kao C.H. (2002). The effect of NaCl on proline accumulation in rice leaves. Plant Growth Regul..

[16] Debouba M., Maaroufi-Dghimi H., Suzuki A., Ghorbel M.H., Gouia H. (2007). Changes in growth and activity of enzymes involved in nitrate reduction and ammonium assimilation in tomato seedlings in response to NaCl stress. Ann. Bot..

[17] Wang Z.Q., Yuan Y.Z., Ou J.Q., Lin Q.H., Zhang C.F. (2007). Glutamine synthetase and glutamate dehydrogenase contribute differentially to proline accumulation in leaves of wheat (*Triticum aestivum*) seedlings exposed to different salinity. J. Plant Physiol..

[18] Zhonghua T., Yanju L., Xiaorui G., Yuangang Z. (2011). The combined effects of salinity and nitrogen forms on *Catharanthus roseus*: The role of internal ammonium and free amino acids during salt stress. J. Plant Nutr. Soil Sci..

[19] Silveira J.A.G., Melo A.R.B., Viégas R.A., Oliveira J.T.A. (2001). Salinity-induced effects on nitrogen assimilation related to growth in cowpea plants. Environ. Exp. Bot..

[20] Nguyen H.T.T., Shim I.S., Kobayashi K., Usui K. (2005). Regulation of ammonium accumulation during salt stress in rice (*Oryza sativa* L) seedlings. Plant Prod. Sci..

[21] Veeranagamallaiah G., Chandraobulreddy P., Jyothsnakumari G., Sudhakar C. (2007). Glutamine synthetase expression and pyrroline-5-carboxylate reductase activity influence proline accumulation in two cultivars of foxtail millet (*Setaria italica* L) with different salt sensitivity. Environ. Exp. Bot..

[22] Kant S., Kant P., Lips H., Barak S. (2007). Partial substitution of NO_3_^−^ by NH_4_^+^ fertilization increases ammonium assimilating enzymes activities and reduces the deleterious effects of salinity on the growth of barley. J. Plant Physiol..

[23] Ashraf M., Foolad M.R. (2007). Roles of glycine betaine and proline in improving plant abiotic stress resistance. Environ. Exp. Bot..

[24] Brown P.H., Bellaloui N., Wimmer M.A., Bassil E.S., Ruiz J., Hu H., Pfeffer H., Dannel F., Römheld V. (2002). Boron in plant biology. Plant Biol..

[25] Bolaños L., Lukaszewski K., Bonilla I., Blevins D. (2004). Why boron?. Plant Physiol. Biochem..

[26] Herrera-Rodríguez M.B., González-Fontes A., Rexach J., Camacho-Cristóbal J.J., Maldonado J.M., Navarro-Gochicoa M.T. (2010). Role of boron in vascular plants and response mechanisms to boron stresses. Plant Stress.

[27] Ruiz J.M., Lopez-Lefebre L.R., Sanchez E., Rivero R.M., García P.C., Romero L. (2001). Preeliminary studies on the influence of boron on the foliar biomass and quality of tobacco leaves subjected to NO_3_^−^ fertilisation. J. Sci. Food Agric..

[28] Camacho-Cristóbal J.J., González-Fontes A. (2007). Boron deficiency decreases plasmalemma H^+^-ATPase expression and nitrate uptake, and promotes ammonium assimilation into asparagine in tobacco roots. Planta.

[29] Redondo-Nieto M., Rivilla R., El-Hamdaoui A., Bonilla I., Bolaños L. (2001). Boron deficiency affects early infection events in the pea-Rhizobium symbiotic interaction. Aust. J. Plant Physiol..

[30] Camacho-Cristóbal J.J., Rexach J., González-Fontes A. (2008). Boron in plants: Deficiency and toxicity. J. Integr. Plant Biol..

[31] Camacho-Cristóbal J.J., Navarro-Gochicoa M.T., Rexach J., González-Fontes A., Herrera-Rodríguez M.B. (2018). Plant response to boron deficiency and boron use efficiency in crop plants. Plant Micronutrients Use Efficiency.

[32] Levene T.R., Hullett C.R. (2002). Eta squared, partial eta squared, and misreporting of effect size in communication research. Hum. Commun. Res..

[33] Hayes J.E., Reid J.R. (2004). Boron tolerance in barley is mediated by efflux of boron from the roots. Plant Physiol..

[34] Miwa K., Fujiwara T. (2010). Boron transport in plants: Co-ordinated regulation of transporters. Ann. Bot..

[35] López-Lefebre L.R., Ruiz J.M., Rivero R.M., García P.C., Sánchez E., Romero L. (2002). Supplemental boron stimulates ammonium assimilation in leaves of tobacco plants (*Nicotiana tabacum* L.). Plant Growth Reg..

[36] Camacho-Cristóbal J.J., González-Fontes A. (1999). Boron deficiency causes a drastic decrease in nitrate content and nitrate reductase activity, and increases the content of carbohydrates in leaves from tobacco plants. Planta.

[37] Cervilla L.M., Blasco B., Ríos J.J., Rosales M.A., Rubio-Wilhelmi M.M., Sánchez-Rodríguez E., Romero L., Ruiz J.M. (2009). Response of nitrogen metabolism to boron toxicity in tomato plants. Plant Biol..

[38] Shen Z., Liang Y.C., Shen K. (1993). Effect of boron on the nitrate reductase activity in oilseed rape plants. J. Plant Nutr..

[39] Ramón A.M., Carpena-Ruiz R.O., Gárate A. (1989). In vitro stabilization and distribution of nitrate reductase in tomato plants. Incidence of boron deficiency. J. Plant Physiol..

[40] Teixeira J., Fidalgo F. (2009). Salt stress affects glutamine synthetase activity and mRNA accumulation on potato plants in an organ-dependent manner. Plant Physiol. Biochem..

[41] Leidi E.O., Lips S.H. (1990). The effect of NaCl salinity on photosynthesis, ^14^C-translocation and yield in wheat plants irrigated with ammonium or nitrate solutions. Irrig. Sci..

[42] Carillo P., Mastrolonardo G., Nacca F., Fuggi A. (2005). Nitrate reductase in durum wheat seedlings as affected by nitrate nutrition and salinity. Funct. Plant Biol..

[43] Hirel B., Martín A., Tercé-Laforgue T., González-Moro M.B., Estavillo J.M. (2005). Physiology of maize I: A comprehensive and integrated view of nitrogen metabolism in a C4 plant. Physiol. Plant.

[44] Gautam S., Singh P. (2009). Salicylic acid-induced salinity tolerance in corn grown under NaCl stress. Acta Physiol. Plant..

[45] Kamachi K., Yamaya T., Hayakawa T., Mae T., Ojima K. (1992). Changes in cytosolic glutamine synthetase polypeptide and its RNA in leaf blade of rice plants during natural senescence. Plant Physiol..

[46] Brugière N., Dubois F., Limami A., Lelandais M., Roux Y., Sangwan R.S., Hirel B. (1999). Glutamine synthetase in the phloem plays a major role in controlling proline production. Plant Cell.

[47] Martin A., Belastegui-Macadam X., Quilleré I., Floriot M., Valadier M.H., Pommel B., Andrieu B., Donnison I., Hirel B. (2005). Nitrogen management and senescence in two maize hybrids differing in the persistence of leaf greenness: Agronomic, physiological and molecular aspects. New Physiol..

[48] Teixeira J., Pereira S., Queirós F., Fidalgo F. (2006). Specific roles of potato glutamine synthetase isoenzymes in callus tissue grown under salinity: Molecular and biochemical responses. Plant Cell Tiss. Organ Cult..

[49] Silveira J.A.G., Viégas R.A., Rocha I.M.A., Monteiro-Moreira A.C.O., Moreira R.A., Oliveira J.O.A. (2003). Proline accumulation and glutamine synthetase activity are increased by salt-induced proteolysis in cashew leaves. J. Plant Physiol..

[50] Pang Q., Chen S., Dai S., Chen Y., Wang Y., Yan X. (2010). Comparative proteomics of salt tolerance in *Arabidopsis thaliana* and *Thellungiella halophila*. J. Proteome Res..

[51] Kumar-Swami A., Alam S.I., Sengupta N., Sarin R. (2011). Differential proteomic analysis of salt response in *Sorghum bicolor* leaves. Environ. Exp. Bot..

[52] Di Martino C., Delfine S., Pizzuto R., Loreto F., Fuggi A. (2003). Free amino acids and glycine betaine in leaf osmoregulation of spinach responding salt stress. New Phytol..

[53] Mansour M.M.F. (2000). Nitrogen containing compounds and adaptation of plants to salinity stress. Biol. Plant..

[54] Hoshida H., Tanaka Y., Hibino T., Hayashi Y., Tanaka A., Takabe T., Takabe T. (2000). Enhanced tolerance to salt stress in transgenic rice that overexpresses chloroplast glutamine synthetase. Plant Mol. Biol..

[55] González-Moro M.B., Loureiro-Beldarrain I., Estavillo J.M., Duñabeitia M.K., Muñoz-Rueda A., González-Murua C. (2003). Effect of photorespiratory C_2_ acids on CO_2_ assimilation, PS II photochemistry and the xanthophyll cycle in maize. Photosynth. Res..

[56] Ranieri A., Bernardi R.M., Lanese P., Soldatini G.F. (1989). Changes in free amino acid content and protein pattern of maize seedlings under water stress. Environ. Exp. Bot..

[57] Wu D., Cai S., Chen M., Ye L., Chen Z., Zhang H., Dai F., Wu F., Zhang G. (2013). Tissue metabolic responses to salt stress in wild and cultivated barley. PLoS ONE.

[58] Berteli F., Corrales E., Guerrero C., Ariza M.J., Pliego F., Valpuesta V. (1995). Salt stress increases ferredoxin-dependent glutamate synthase activity and protein level in the leaves of tomato. Physiol. Plant..

[59] Lutts S., Majerus V., Kinet J.M. (1999). NaCl effects on proline metabolism in rice (*Oryza sativa*) seedlings. Physiol. Plant..

[60] Ferguson L., Poss J.A., Grattan S.R., Grieve C.M., Wang D., Wilson C., Donovan T.J., Chao C.T. (2002). Pistachio rootstocks influence scion growth and ion relations under salinity and boron stress. J. Am. Soc. Hortic. Sci..

[61] Grattan S.R., Shannon M.C., Grieve C.M., Poss J.A., Suarez D.L., Leland F. (1996). Interactive effects of salinity and boron on the performance and water use of eucalyptus. Acta Hortic..

[62] Holloway R.E., Alston A.M. (1992). The effects of salt and boron on growth of wheat. Aust. J. Agric. Res..

[63] Grieve C.M., Poss J.A. (2000). Wheat response to interactive effects of boron and salinity. J. Plant Nutr..

[64] Alpaslan M., Gunes A. (2001). Interactive effects of boron and salinity stress on the growth, membrane permeability and mineral composition of tomato and cucumber plants. Plant Soil.

[65] Wimmer M.A., Bassil E.S., Brown P.H., Läuchli A. (2005). Boron response in wheat is genotype-dependent and related to boron uptake, translocation, allocation, plant phenological development and growth rate. Funct. Plant Biol..

[66] Huanca-Mamani W., Arias-Carrasco R., Cárdenas-Ninasivincha S., Rojas-Herrera M., Sepúlveda-Hermosilla M., Caris-Maldonado J.C., Bastías E., Maracaja-Coutinho V. (2018). Long non-coding RNAs responsive to salt and boron stress in the hyper-arid Lluteño maize from Atacama Desert. Genes.

[67] González-Moro M.B., Lacuesta M., Royuela M., Muñoz-Rueda A., González-Murua C. (1993). Comparative study of the inhibition of photosynthesis caused by aminooxyacetic acid and phosphinothricin in *Zea mays*. J. Plant Physiol..

[68] Cataldo D.A., Maroon M., Schrader L.E., Youngs V.L. (1975). Rapid colorimetric determination of nitrate in plant tissue by nitrification of salicylic acid. Comm. Soil Sci. Plant Anal..

[69] O’Neal D., Joy K.W. (1973). Glutamine synthetase of pea leaves. I. Purification, stabilization and pH optima. Arch. Biochem. Biophys..

[70] Bradford M.M. (1976). A rapid and sensitive method for the quantitation of microgram quantities of protein utilizing the principle of protein-dye binding. Anal Biochem..

[71] Laemmli U.K. (1970). Cleavage of structural proteins during the assembly of the head of bacteriophage T4. Nature.

